# Infarctus du myocarde inférieur: première série marocaine, à propos de 103 cas

**DOI:** 10.11604/pamj.2019.33.74.16047

**Published:** 2019-05-31

**Authors:** Fatima-Zahra El Hattab, Fatima Zohra Radi, Loubna Hara, El Mehdi Hafidi, Jamila Zarzur, Mohamed Cherti

**Affiliations:** 1 Service de Cardiologie B, Université Mohamed V Souissi, Rabat, Maroc

**Keywords:** Infarctus du myocarde inférieur, angioplastie coronaire, complications, Inferior myocardial infarction, coronary angioplasty, complications

## Abstract

L'infarctus du myocarde (IDM) représente une cause majeure de mortalité cardiovasculaire. L'IDM inférieur représente 30 à 50% de l'ensemble des infarctus avec un pronostic favorable par rapport à l'infarctus antérieur. Le but de notre travail est d'étudier les aspects épidémiologiques, cliniques, électriques, échocardiographiques et angiographiques de l'IDM inférieur, ainsi que ses complications et ses modalités thérapeutiques. Sur une période de 3 ans, nous avons admis 720 patients pour *ST-Elevation Myocardial Infarction* (STEMI) dont 103 de topographie inférieure soit une fréquence de 14,3%. On note une nette prédominance masculine avec une moyenne d'âge de 58 ans pour les hommes et 62 ans pour les femmes. Le tabagisme représente le principal facteur de risque cardiovasculaire retrouvé dans 57,28% des cas. L'infarctus ventricule droit (VD) est objectivé chez 11,65% des malades. La moitié de ces patients ont présenté une instabilité hémodynamique. Le *Bicuspid aortic valve* (BAV) 3^ème^ degré a été diagnostiqué chez 12,6% des patients. Sur le plan thérapeutique, sept malades ont été thrombolysés et 42 ont bénéficié d'une coronarographie. La lésion coupable de l'IDM inférieur était la coronaire droite dans 53% des cas et l'artère circonflexe dans 47%. La coronaire droite est responsable de l'infarctus VD dans 100% des cas. L'angioplastie coronaire est réalisée chez 18 patients dans la suite de la coronarographie. Onze malades ont bénéficié d'une angioplastie transluminale (ATL) de la coronaire droite et celle de la circonflexe est réalisée chez 2 malades. La mortalité précoce à 30 jours est estimée à 1,94%. Dans le groupe de patients présentant un infarctus VD, la mortalité est aux alentours de 17%.

## Abstract (en)

Myocardial infarction (MI) is a major cause of cardiovascular mortality. Inferior MI accounts for 30-50% of infarctions but it is associated with a favorable prognosis compared to anterior infarct. This study aimed to study the epidemiological, clinical, electrical, echocardiographic, angiographic features of inferior MI, as well as its complications and its therapeutic approaches. Over a period of 3 years, we admitted 720 patients with STEMI, of whom 103 with inferior STEMI, reflecting a rate of 14.3%. There was a clear male predominance, with an average age of 58 years (men) and 62 years (women). Smoking was the main risk factor for cardiovascular disease (57.28% of patients were smokers). Right ventricular infarction was found in 11.65% of cases. Half of these patients had hemodynamic instability. Third-degree atrioventricular block was diagnosed in 12.6% of patients. Therapeutic approach was based on thrombolysis (7 patients) and coronary angiogram (42 patients). Inferior MI was caused by right coronary lesion in 53% of cases and circumflex artery occlusion in 47% of cases. Right coronary is responsible for right ventricular infarction in 100% of cases. Coronary angioplasty was performed in 18 patients after coronary angiogram. Eleven patients underwent transluminal coronary angioplasty in the right coronary while 2 patients underwent transluminal coronary angioplasty in the circumflex artery. Early mortality at 30 days was 1.94%. In the right ventricular infarction group mortality rate was about 17%.

## Introduction

La pathologie coronaire constitue un problème majeur de santé publique dans le monde avec un taux alarmant de 12,8% [1]. L'infarctus du myocarde (IDM) représente une cause majeure de mortalité cardiovasculaire. Il est en nette progression dans les pays en voie de développement en raison de la recrudescence des facteurs de risque cardiovasculaires. L'IDM inférieur représente 30 à 50% de l'ensemble des infarctus avec un pronostic favorable par rapport à l'infarctus antérieur [2]. Il est caractérisé par ses présentations cliniques très diverses et par des complications; qui lui sont propres; comme les troubles conductifs et l'infarctus du ventricule droit. Il est important de noter que ces complications sont présentes chez près de 50% des patients ayant un infarctus inférieur et sont associées à une mortalité accrue, ce qui transforme significativement le pronostic favorable. A travers ce travail, nous allons étudier les aspects épidémiologiques, cliniques, électriques, échocardiographiques et angiographiques de l'IDM inférieur, ainsi que ses complications et ses modalités thérapeutiques.

## Méthodes

Il s'agit d'une étude rétrospective descriptive, menée sur une période de 3 ans s'étalant de janvier 2014 à décembre 2016. Nous avons colligé 103 patients hospitalisés pour IDM de topographie inférieure au Service de Cardiologie B de la maternité Souissi de Rabat. Les patients hospitalisés pour IDM circonférentiel ou septal profond et les patients avec antécédent de cardiopathie ischémique ont été exclus de notre étude. L'analyse rétrospective a été faite en recueillant les données à partir du registre du Service de Cardiologie B et des dossiers cliniques des malades. On a rapporté les différents paramètres sur des fiches d'exploitation.

## Résultats

Entre janvier 2014 et décembre 2016, 720 patients ont été admis au service de cardiologie B pour un Syndrome coronarien aigu (SCA) avec sus décalage permanent du segment ST dont 103 sont de topographie inférieure, soit une fréquence de 14,3%. Les hommes constituent 79,62% de la population étudiée, alors que la fréquence des femmes est de 20,38% avec un sex ratio H/F de 3,9. La moyenne d'âge est de 58 ans pour les hommes et 62 ans pour les femmes. Le pic de fréquence se situe entre 61 et 70 ans. Le tabagisme représente le principal facteur de risque cardiovasculaire retrouvé dans 57,28 % des cas. Le diabète et l'hypertension artérielle (HTA) sont présents respectivement chez 37,86% et 25,24% des malades. Le signe d'appel majeur était la douleur. Les symptômes accompagnateurs étaient présents chez la moitié des patients. Un seul malade a présenté un épisode de syncope et 8 malades des lipothymies, soit respectivement 0,97% et 7,77% des cas. Concernant le délai de consultation, 72% des patients ont consulté 12 heures après le début de la symptomatologie. L'instabilité hémodynamique a été retrouvée dans 5,8% des cas. Les signes d'insuffisance cardiaque droite étaient présents chez 23,3% des patients, tandis que 7,77% étaient en insuffisance cardiaque gauche. Nous avons réparti les malades en 2 groupes: patients avec infarctus VD (groupe 1) et patients sans infarctus VD (groupe 2) puis on a étudié la fréquence de l'instabilité hémodynamique, l'insuffisance cardiaque droite et l'insuffisance cardiaque gauche chez les deux groupes. Les résultats figurent dans le Tableau 1. L'electrocardiogramme (ECG), fait chez tous les malades à l'admission avec réalisation systématique des dérivations droites, a montré un sus décalage du ST en inférieur isolé chez 79,7% des malades. L'infarctus inféro-basal et inféro-latéral bas ont été retrouvés respectivement chez 8,73% et 6,8% des cas. L'infarctus VD n'a été documenté électriquement que chez 4,9% des malades et cela est expliqué par le retard de consultation de la majorité de nos malades ce qui rend la probabilité d'objectiver le sus décalage du ST dans les dérivations droites faible au-delà des premières 24h. L'arythmie complète par fibrillation atriale (ACFA) est retrouvée chez 5% des patients. Trente-neuf malades de notre série, soit 37,8% des cas, ont présenté un BAV. Le BAV 3^ème^degré a été diagnostiqué chez 12,6% des patients. L'échocardiographie Doppler était réalisée chez tous les patients de notre série. Une dilatation des cavités droites a été retrouvée chez 07 patients soit 6,8% de la population étudiée. Les anomalies de la contractilité segmentaire de la paroi libre du VD étaient présentes chez 11,65% des patients. Dans notre série, la a fraction d'éjection du ventricule gauche (FEVG) varie entre 20% et 65%. Concernant les troubles de la cinétique segmentaire du VG, la paroi inférieure était akinétique dans 65% suivie de la paroi inférolatérale dans 41% des cas. Deux malades ont présenté un épanchement péricardique. L'insuffisance mitrale a été objectivée chez 05 patients. La fuite est jugée sévère chez 2 malades. La troponine demandée chez tous nos malades est revenue positive. Le bilan rénal a objectivé une insuffisance rénale fonctionnelle dans 14% des cas. La clearance de créatinine était moins de 60 ml/min chez 29% de nos patients.

**Tableau 1:** Répartition de la fréquence de l’instabilité hémodynamique, l’insuffisance cardiaque droite et gauche chez les deux groupes de patients

	Groupe 1 (n=12)	Groupe 2 (n=91)
***Instabilité hémodynamique***	50%	0%
***IC gauche***	16,7%	6,6%
***IC droite***	100%	13%

**Figure 1:**
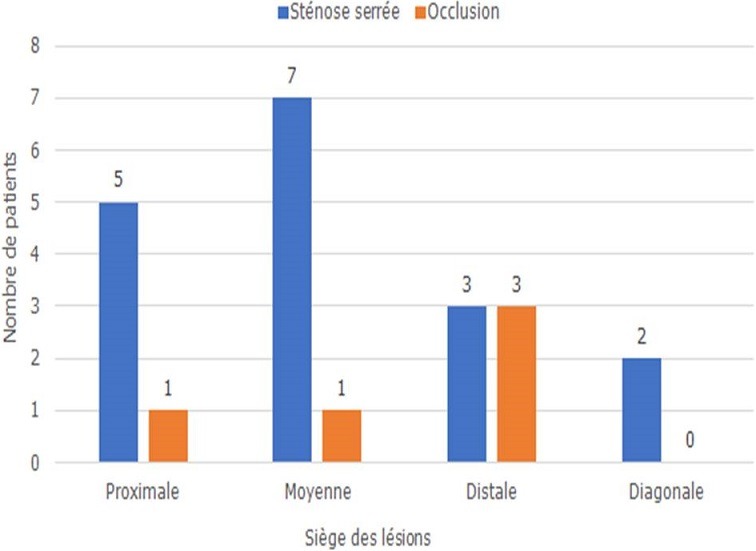
Répartition des sténoses serrées et des occlusions de l’artère interventriculaire antérieure selon leur siège

**Figure 2:**
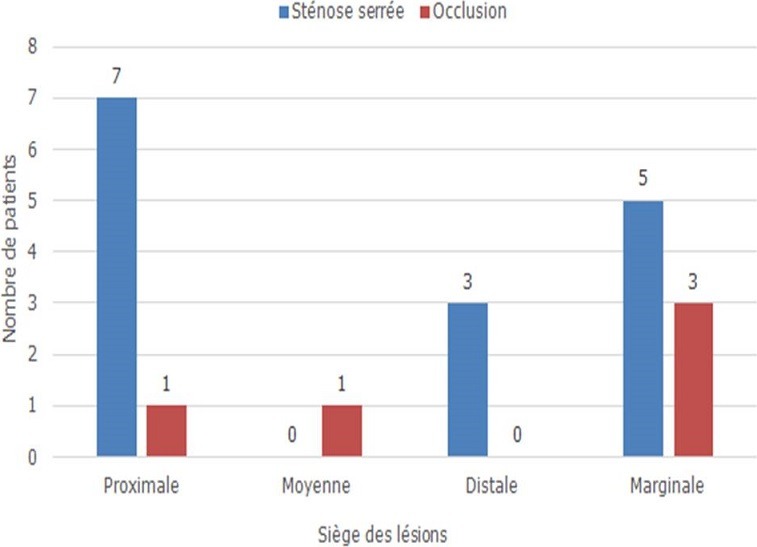
Répartition des sténoses serrées et des occlusions de l’artère circonflexe selon leur siège

**Figure 3:**
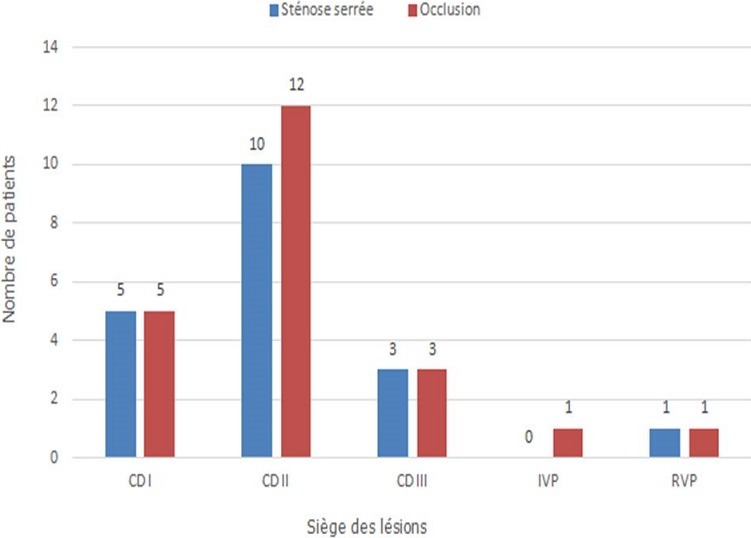
Répartition des sténoses serrées et des occlusions de la coronaire droite selon leur siège

**Figure 4:**
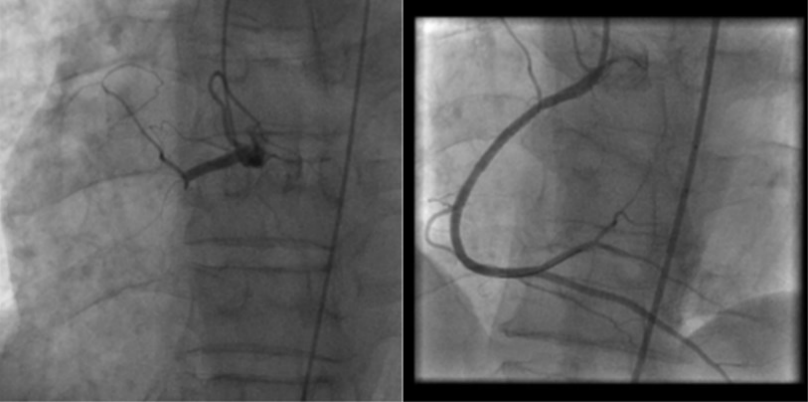
Coronarographie montrant une occlusion de la coronaire droite à la partie initiale de son 2^ème^segment et le résultat angiographique après désobstruction

**Figure 5:**
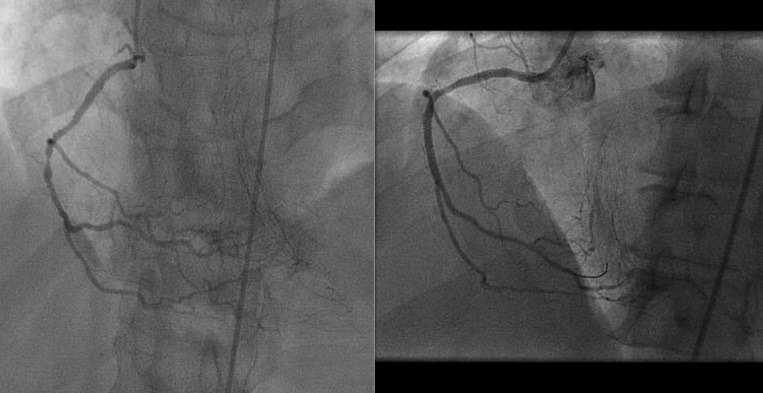
Coronarographie objectivant une lésion serrée de la partie moyenne de la coronaire droite et résultat après angioplastie

Sur le plan thérapeutique, sept malades ont été thrombolysés. Tous les patients ont reçu la double anti-agrégation plaquettaire, l'héparinothérapie à base d'HBPM et une statine. Six malades ont nécessité un remplissage vasculaire et le recours à la dobutamine a été indiqué chez 2 malades. L'atropine a été utilisée chez 2 patients. L'inhibiteur de l'enzyme de conversion et le ' bloqueur ont été prescris respectivement chez 75 et 69 patients. Quarante-deux patients ont subi une coronarographie. La répartition des sténoses serrées et des occlusions de l'artère interventriculaire antérieure, la circonflexe et la coronaire droite est représentée respectivement dans les Figure 1, Figure 2, Figure 3. La lésion coupable de l'IDM inférieur était la coronaire droite dans 53% des cas et l'artère circonflexe dans 47%. La coronaire droite était responsable de l'infarctus VD dans 100% des cas. L'angioplastie coronaire a été réalisée chez 18 patients dans la suite de la coronarographie. Seuls 13 malades ont bénéficié d'une ATL de l'artère coupable dans la suite de la coronarographie, soit 12,62 % des cas. Onze malades ont subi une ATL de la coronaire droite (Figure 4, Figure 5) et celle de la circonflexe a été réalisée chez 2 malades. A noter que 5 de nos malades ont bénéficié d'une ATL de l'artère interventriculaire antérieure (IVA), malgré qu'en aucun cas l'IVA n'a été l'artère coupable de l'IDM inférieur, mais elle était siège de lésions proximales ou moyennes très serrées. Pour les autres malades, on a complété par un test de viabilité ou d'ischémie pour prendre une décision thérapeutique. On a opté pour un traitement médical pour 2 malades. Deux patients ont bénéficié d'une montée de sonde d'entrainement électrosystolique. La mortalité précoce à 30 jours est estimée à 1,94%. Dans le groupe de patients présentant un infarctus VD, la mortalité était aux alentours de 17%.

## Discussion

L'IDM constitue une urgence cardiologique absolue dont l'incidence reste encore élevée avec 120 000 cas par an en France [3]. Son pronostic reste grave puisqu'il est responsable de 10 à 12% de la mortalité totale annuelle chez l'adulte en France [4, 5]. Dans notre étude, seuls les patients ayant présenté un SCA avec sus décalage persistant de topographie inférieure ont été étudiés. Les IDM inférieurs représentent 30 à 50 % de l'ensemble des IDM et sont, en général, de bon pronostic par rapport aux antérieurs [2]. La fréquence retrouvée dans notre travail est de 14,3%. La moyenne d'âge de nos patients est de 60 ans et la tranche d'âge prédominante est entre 61 et 70 ans. Ce résultat est similaire à celui retrouvé dans l'étude menée par Pascal et al [6] en 1988 et par Chen et al en 2011 [7]. La moyenne d'âge, dans l'étude turque faite par Uluganyan et al, est de 57+/-11,5 ans [8]. On a noté une nette prédominance masculine avec sex ratio H/F de 3,9, donnée concordante avec la littérature [6-8] (Tableau 2). Le tabagisme représente le principal facteur de risque cardiovasculaire chez notre population, présent dans 59% des cas. Ce constat est proche de celui objectivé par Pascal *et al* [6]. Les études indienne et chinoise ont trouvé une fréquence moins importante de, respectivement, 45% et 31% [7, 9]. Le diabète est retrouvé chez 39% de nos malades. Cette fréquence est la plus élevée par rapport aux séries de la littérature [6, 7, 9]. La prévalence des patients hypertendus dans les études de Pascal [6], Chen [7] ainsi que l'étude de Daanish [9] était au alentour de 50%, résultat discordant avec le mien. Plusieurs séries indiennes se sont intéressées à la recherche de l'infarctus du VD chez les patients ayant présenté un SCA avec sus décalage persistant de topographie inférieure. Ils ont répartis leurs malades en deux groupes: Patients avec infarctus VD (groupe 1) et patients sans infarctus VD (groupe 2), puis ils ont étudié la prévalence de l'instabilité hémodynamique, l'insuffisance cardiaque droite et gauche chez les deux groupes. Le Tableau 3 représente la fréquence des 3 formes cliniques dans les 2 groupes de patients selon la littérature [9-11].

**Tableau 2:** Comparaison de la répartition des patients selon le sexe entre notre série et celles de la littérature

Séries	Male	Female
***Pascal et al (1988)***	76%	23.9%
***Chen et al (2001)***	82%	18%
***Uluganyan et al (2016)***	75.4%	24.5%
**Notre série**	**79.6%**	**20.38%**

**Tableau 3:** Fréquence de l’instabilité hémodynamique, l’IC droite et gauche chez les 2 groupes selon les séries de la littérature

Séries	Instabilité hémodynamique		IC droite		IC gauche	
	*Groupe 1*	*Groupe 2*	*Groupe 1*	*Groupe 2*	*Groupe 1*	*Groupe 2*
***Daanish et al [******9******]***	56.25%	11.86%	43.7%	0%	16.7%	11.86%
***Garg et al [******11******]***	21%	7.5%	21.2%	3%	18%	17%
***Achutoch et al [******10******]***	-	-	77%	0%	-	-
**Notre série**	**50%**	**0%**	**100%**	**13%**	**16.7%**	**6.6%**

IC: Insuffisance cardiaque

Les troubles conductifs constituent une complication assez fréquente des IDM inférieurs. Nous avons objectivé un BAV complet dans 12,6% des cas. Ce résultat rejoint les séries de Jewitt, Julian et Tans [12-14]. Concernant la fibrillation atriale, notre fréquence est proche de Pascal et al [6]. L'infarctus VD est la complication la plus redoutable de l'IDM inférieur. Il a été objectivé chez 11,65% des patients de notre série. D'après les séries de la littérature, sa prévalence varie entre 22 et 55%. Ashutosh et al [10] et Overgaard *et al*[15] ont mentionné respectivement une prévalence de 22 et 23,7%. Les chiffres les plus importants (50%) ont été rapportés par l'équipe de Klein et al dans leur travail destiné à évaluer la prévalence de cette entité [16]. Sur le plan angiographique, la coronaire droite est l'artère coupable dans 53% des cas ce qui est comparable avec la littérature [7, 8]. Nous avons documenté une mortalité hospitalière précoce de 2% ce qui est parfaitement similaire au résultat de Daanish et al [9]. Le taux de mortalité communiqué par les autres auteurs varie entre 3,7 et 8,5% [6-8, 17]. Malgré que nous avons rapporté dans notre étude le taux de mortalité globale le plus bas, le taux objectivé au sein du groupe infarctus VD était surprenant : 17% des malades appartenant à ce groupe sont décédés. En ce qui concerne ce dernier constat, on est loin de la littérature puisque Daanish et al [9] parlent d'un taux de 6% et Overgaard et al de 8,1% [15].

## Conclusion

L'IDM, quel que soit sa topographie, constitue une urgence cardiologique absolue. L'IDM inférieur est caractérisé par des complications, représentées essentiellement par l'infarctus VD et les troubles conductifs, pouvant mettre en jeu le pronostic vital et transformer par conséquent son pronostic favorable. La consultation précoce ainsi qu'une meilleure connaissance de ces complications permettent une prise en charge rapide et adéquate afin de réduire les taux de mortalité élevés objectivés chez notre population.

### Etat des connaissances actuelles sur le sujet

- Il est de bon pronostic par rapport à l'infarctus antérieur;
- Touche généralement le sujet jeune;
- L'infarctus inférieur se caractérise par ses complications: l'extension VD, le BAV.

### Contribution de notre étude à la connaissance

- Un taux de mortalité élevé dans les IDM inférieur avec extension VD comparativement à la littérature.

## Conflits des intérêts

Les auteurs ne déclarent aucun conflit d’intérêts.
